# Engineered peptide PLG0206 overcomes limitations of a challenging antimicrobial drug class

**DOI:** 10.1371/journal.pone.0274815

**Published:** 2022-09-16

**Authors:** David B. Huang, Kimberly M. Brothers, Jonathan B. Mandell, Masashi Taguchi, Peter G. Alexander, Dana M. Parker, Dean Shinabarger, Chris Pillar, Ian Morrissey, Stephen Hawser, Parviz Ghahramani, Despina Dobbins, Nicholas Pachuda, Ronald Montelaro, Jonathan D. Steckbeck, Kenneth L. Urish

**Affiliations:** 1 Peptilogics, Pittsburgh, Pennsylvania, United States of America; 2 Department of Orthopedic Surgery, Arthritis and Arthroplasty Design Group, University of Pittsburgh, Pittsburgh, Pennsylvania, United States of America; 3 Department of Orthopedic Surgery, Tokyo Women’s Medical University, Medical Center East, Tokyo, Japan; 4 Micromyx, Kalamazoo, Michigan, United States of America; 5 IHMA, Monthey, Switzerland; 6 Inncelerex, Jersey City, New Jersey, United States of America; 7 Department of Microbiology and Molecular Genetics, University of Pittsburgh, Pittsburgh, Pennsylvania, United States of America; 8 The Bone and Joint Center, Magee Women’s Hospital of the University of Pittsburgh Medical Center, Pittsburgh, Pennsylvania, United States of America; 9 Department of Bioengineering, and Clinical and Translational Science, University of Pittsburgh, Pittsburgh, Pennsylvania, United States of America; Laurentian University, CANADA

## Abstract

The absence of novel antibiotics for drug-resistant and biofilm-associated infections is a global public health crisis. Antimicrobial peptides explored to address this need have encountered significant development challenges associated with size, toxicity, safety profile, and pharmacokinetics. We designed PLG0206, an engineered antimicrobial peptide, to address these limitations. PLG0206 has broad-spectrum activity against >1,200 multidrug-resistant (MDR) ESKAPEE clinical isolates, is rapidly bactericidal, and displays potent anti-biofilm activity against diverse MDR pathogens. PLG0206 displays activity in diverse animal infection models following both systemic (urinary tract infection) and local (prosthetic joint infection) administration. These findings support continuing clinical development of PLG0206 and validate use of rational design for peptide therapeutics to overcome limitations associated with difficult-to-drug pharmaceutical targets.

## Introduction

Antimicrobial resistance is a worldwide public health threat [1] and there is a lack of new agents in the antibiotic discovery pipeline [2,3]. Multidrug-resistant (MDR) organisms are associated with hospital-acquired infections and include *Enterococcus faecium*, *Staphylococcus aureus*, *Klebsiella pneumoniae*, *Acinetobacter baumannii*, *Pseudomonas aeruginosa*, *Enterobacter* spp., and *Escherichia coli* (ESKAPEE pathogens). It has been almost 40 years since the last new class of broad-spectrum antimicrobials, carbapenems, was introduced [4]. Given the high morbidity and mortality of these infections [5–8], economic cost [9], and growing antibiotic resistance [10,11], new solutions are needed.

**Fig 1:**
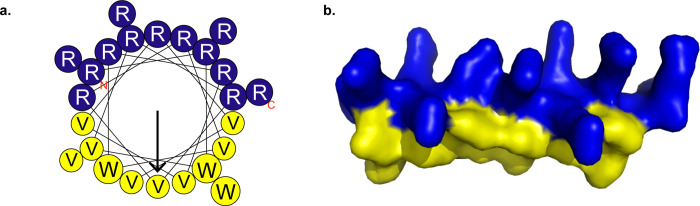
The primary sequence and structural models of PLG0206. Primary sequence: H-RRWVRRVRRVWRRVVRVVRRWVRR-NH2. Structural models: (**a**) helical wheel water-excluding surface; (**b**) diagram 3D helical structural model. Fig 1A was reproduced (with changes) from Fig 1 of Deslouches et al. *Antimicrob Agents Chemother* 59, 1329–1333 (2015) [19] which is covered by Creative Commons Attribution 4.0 license. 3D, three-dimensional; R, arginine; V, valine; W, tryptophan.

Antimicrobial peptides (AMPs) are a molecular class that occurs naturally as part of the intrinsic defense mechanisms of numerous species. The clinical development of AMPs has been limited by several factors, including toxicity, suboptimal pharmacokinetic (PK) properties, and lack of robust in vivo activity [12–14]. To overcome these limitations, we have designed and continue to develop an engineered synthetic antibacterial peptide, PLG0206 (previously referenced as WLBU2). PLG0206 is a 24-amino-acid peptide containing only arginine, valine, and tryptophan residues sequenced to maximize bacterial membrane binding and interaction while minimizing the potential for toxicity that is associated with the broader class [12,15] (Fig 1). Previous studies have demonstrated that PLG0206 has potential broad-spectrum activity [12,15], potent *S*. *aureus* biofilm eradication [16,17], and a low propensity for emergence of resistance in *P*. *aeruginosa* [18].

Based on these early limited findings, we hypothesized that PLG0206 could be effective as a broad-spectrum antimicrobial for infections caused by MDR bacteria. Here, we detail the early pre-clinical development of PLG0206 from in vitro to in vivo proof-of-concept studies that support an active antibacterial agent that overcomes many of the limitations associated with current experimental and commercial antibiotics. In this article, we show PLG0206 has rapid bactericidal, broad-spectrum activity against ESKAPEE MDR pathogens, and importantly is active against both planktonic and biofilm growth states. The efficacy of PLG0206 in treating infections in vivo was demonstrated with biofilm-based infections in a *S*. *aureus* large animal model of periprosthetic joint infections (PJI) and a murine model of uropathogenic *E*. *coli*. Across numerous animal studies, PLG0206 has demonstrated a low toxicity profile for both local and systemic use. These findings support the clinical development of PLG0206 and reinforce the potential utility of peptides as therapeutic design substrates to overcome limitations of different pharmaceutical classes.

## Materials and methods

### Bacterial strains and growth conditions

For in vitro assays, bacterial strains tested were reference strains from the American Type Culture Collection (Manassas, VA) or clinical isolates from the IHMA collection. The IHMA collection contained clinical isolates collected in 2019 from various hospitals worldwide. Prior to testing, all isolates were streaked from frozen vials onto trypticase soy agar (TSA) plates with 5% sheep blood (Liofilchem, Teramo, Italy) and incubated overnight at 35°C.

### Determination of minimum inhibitory concentration

Broth dilution MICs of PLG0206 and the comparators ceftazidime, ceftolozane–tazobactam, clindamycin, colistin, daptomycin, doxycycline, levofloxacin, linezolid, meropenem, piperacillin–tazobactam, tobramycin, trimethoprim–sulfamethoxazole, and vancomycin were determined in accordance with guidelines from the Clinical and Laboratory Standards Institute [20,21]. The medium employed for testing the comparator antibiotics in the broth microdilution minimum inhibitory concentration (MIC) assay for all organisms was cation-adjusted Mueller–Hinton Broth (CAMHB; Becton Dickinson, Franklin Lakes, NJ). Due to poor solubility in CAMHB, PLG0206 broth microdilution testing utilized RPMI-1640 medium (Sigma Aldrich, Saint Louis, MO) with MOPS (Sigma Aldrich; Saint Louis MO). Both mediums were supplemented with 0.002% P-80 (EMD Millipore, Burlington, MA). Due to a complete lack of activity in Mueller-Hinton Agar (S1 Table), PLG0206 agar dilution MIC testing for the spontaneous mutation frequency assay was conducted using Roswell Park Memorial Institute 1640 (RPMI) broth and 1.5% high EEO agarose (RPMI-agarose).

### Planktonic time-kill studies

The inoculum was prepared from growth on a freshly streaked plate to equal the turbidity of a 0.5 McFarland standard in phosphate-buffered saline (PBS; pH 7.4), and a portion was transferred to a 96 deep-well plate to achieve a final concentration of 0.5 to 1×10^7^ colony-forming unit (CFU) per mL. The wells contained PLG0206 at a final concentration of 125 or 500 μg/mL, i.e., 2× or 8× the MIC. Levofloxacin was tested as a comparator at 16× the MIC, with the exception of isolates where the MIC was >8 μg/mL, in which the test concentration was set at 128 μg/mL. The positive growth control PBS (pH 7.4) contained no drug. During the time-kill assay, the 96 deep-well plate was left on the bench top at room temperature and wells were sampled for enumeration at 0, 5, 15, 30, and 60 min. Aliquots were removed over time from each well, neutralized by serial dilution in double-strength Dey/Engley broth, and spread onto agar plates for 15 h at 35°C. The resulting colonies were counted, and the CFU/mL was determined from the average count of the duplicate plates. The limit of detection for the assay was 200 CFU/mL. Viable counts (log_10_ CFU/mL) were then plotted versus time.

### Spontaneous mutation frequency

Due to the poor solubility of PLG0206 in Mueller-Hinton broth and inactivity of PLG0206 in standard agar media (S1 Table), agar dilution MIC testing media [21], and spontaneous mutation agar plates were prepared using Roswell Park Memorial Institute 1640 (RPMI) broth and 1.5% high EEO agarose (RPMI-agar). Using the agar dilution MIC values, the appropriate amount of 40× drug was mixed with RPMI-agar (for PLG0206) or Mueller–Hinton agar (for the comparators) for each drug concentration. The 150×15-mm round plates (VWR; Radnor, PA) were prepared using 50 mL of molten agar in duplicate at 4×, 8×, and 16× the MIC for PLG0206 and the comparators. To inoculate the plates, each test isolate was streaked onto two TSA with 5% sheep blood plates, which were incubated for 20 h at 35°C in an ambient atmosphere. Growth from these plates was harvested with sterile cotton swabs and used to prepare a dense cell suspension (2.5×10^9^ to 2.5×10^10^ CFU/mL) in ~5 mL of saline, which constituted the inoculum for the spontaneous mutation plates. For each test plate in duplicate, 0.2 mL of the inoculum was placed on the agar surface and an L-shaped spreader was used to evenly distribute the inoculum, targeting ~10^9^ CFU per 150×15 mm plate. The viable count of each suspension was determined by plating serial 10-fold dilutions onto duplicate agar plates composed of the appropriate medium for each isolate. Once the inoculum was completely absorbed by the agar, the plates were inverted and incubated at 35°C in ambient air for 48 h prior to reading.

Using a sterile toothpick, growth from select spontaneous mutant colonies and smears (PLG0206 only) were transferred to agar plates containing the same drug concentration present during initial spontaneous mutant selection. The identity of the putative spontaneous mutants was confirmed using a MALDI Biotyper (Bruker, Billerica, MA). Select spontaneous mutants were prepared and frozen at −80°C with a cryoprotectant and later evaluated in broth microdilution MIC testing with PLG0206 and several comparator antibiotics [20,21].

### Anti-biofilm activity

ESKAPEE pathogens (*E*. *faecium*, *n* = 18; *S*. *aureus*, *n* = 18; *K*. *pneumoniae*, *n* = 18; *A*. *baumannii*, *n* = 18; *P*. *aeruginosa*, *n* = 12; *E*. *cloacae*, *n* = 18; and *E*. *coli*, *n* = 18), which were MDR, were selected from a large clinical library of MDR and non-MDR organisms. Strains were diluted in Mueller–Hinton Broth (MHB; Becton Dickinson and Company) to a final concentration of 1×10^6^ CFU/mL using the 0.5 MacFarland Standard (GFS Chemicals) and an Infinite M200 Spectrophotometer (Tecan). Implant material was prepared from 0.6-mm diameter stainless steel Kirschner wire (Sklar Instruments #501438322) and cut into 6-mm length, autoclaved, and plated in wells along with ESKAPEE pathogen isolates at 1×10^6^ CFU/mL. After plating, fresh MHB media (Becton Dickinson and Company #212322) was exchanged after 24 h. At 48 h, wires with mature biofilm were washed in 1 mL of Dulbecco’s PBS (dPBS) (Gibco #14190–144) and placed into a solution of 1 mg/mL PLG0206 dissolved into PBS and pH adjusted to 7.4 with 1% sodium hydroxide. Implant pieces were treated for 5, 15, and 30 min. After treatment, they were placed in 1 mL sonication fluid consisting of 1% Tween 20 in double-strength Dey-Engley neutralizing broth (Sigma #D3435) and sonicated for 10 min. The sonicate was serially diluted and plated on TSA II with 5% sheep blood CS100 (Remel #R01202) plates for CFU analysis. Reduction in CFUs was determined from the average of CFUs from untreated Kirschner wires of 48 h biofilms and after a dPBS wash prior to placing in PBS containing 1% Tween 20 (PBST) and sonicating for 10 min. After sonication, treated implant pieces were removed and placed back into fresh MHB under sterile conditions and incubated for 15 h at 37°C. Wells with implant pieces that were turbid displayed remaining biofilm after treatment, whereas clear wells displayed implant pieces that were rendered culture sterile. Data from 18 isolates per pathogen were pooled and graphed as mean standard deviation.

### Rabbit PJI model

Approved by the Institutional Animal Care and Use Committee (IACUC) protocol #20057438, rabbit animal studies were performed at the University of Pittsburgh according to institutional animal care and use policies. Male New Zealand white rabbits weighing 1.5–2.5 kg, experimentally naïve, and 15–17 weeks old (Envigo; Denver, PA), were used for all studies. Males were selected as it was considered that there are no significant anatomical differences between males and females of the surgical site (knee area) and males are larger than females. Animal sample size was determined by power analysis using G*Power version 3.1. Because all animals had similar weights, animals were assigned to study groups chronologically according to US Department of Agriculture identifiers. All animals received equal care and Division of Laboratory Animal Resources staff were masked from knowing which animals were in each treatment group. Only the study director was aware of the group allocation.

### PJI rabbit surgical models

**Fig 2:**
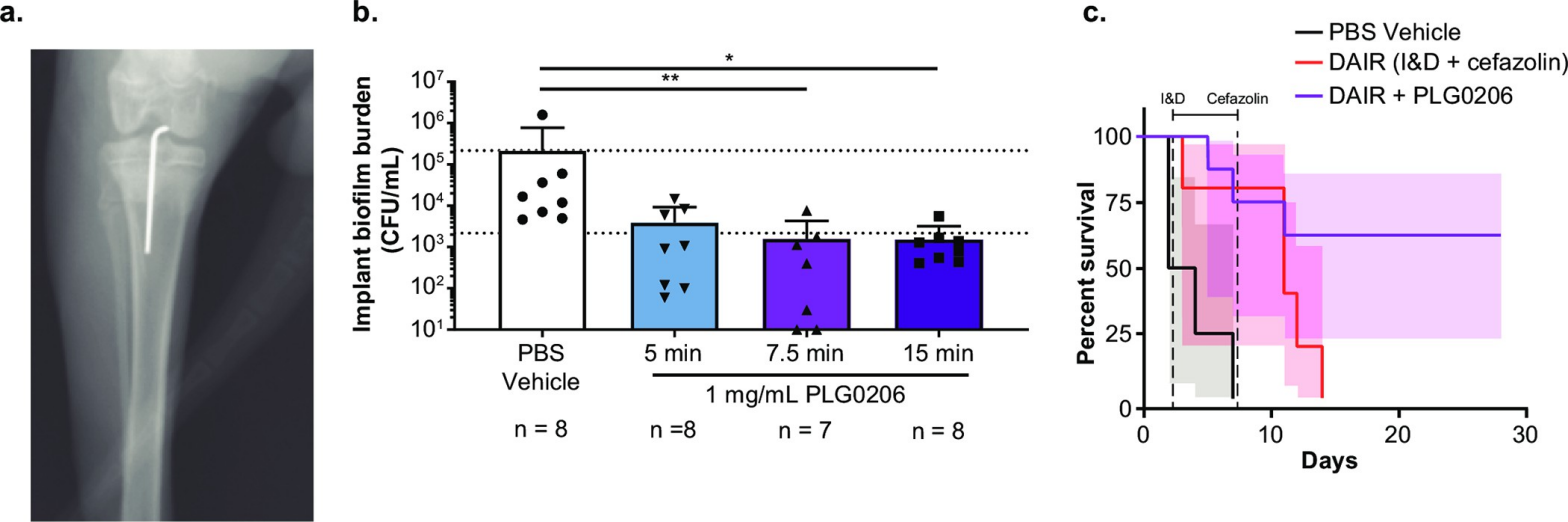
PLG0206 is effective at treating PJI intraoperatively. (a) X-ray verification of Kirschner wire implant into the rabbit tibial canal. (b) In vivo CFU analysis of PLG0206 efficacy using 1 mg/mL PLG0206 to treat PJI. Data are mean (standard deviation). Kruskal–Wallis statistical analysis was performed using GraphPad Prism version 9. *P<0.01 and **P<0.05. (c) Survival analysis of PBS vehicle (I&D alone), DAIR (PBS vehicle + cefazolin), and DAIR+ PLG0206 was performed over 28 days. Shaded region indicates 95% confidence intervals, and no overlap indicates statistical significance (*P*<0.05). CFU, colony-forming units; DAIR, debridement and antibiotics with implant retention; I&D, irrigation and debridement; PBS, phosphate-buffered saline; PJI, periprosthetic joint infection.

Rabbit PJI surgical models were performed according to Yagi et al (2021) [22]. Rabbits were anesthetized with 20 mg/kg ketamine, 2 mg/kg xylazine, and 1–3% isoflurane inhalation. Animals were intubated with a 3 mm endotracheal tube. The left hind limb was shaved and prepared using hanging leg preparation with chlorhexidine scrub and betadine solution. The rabbits were placed in dorsal recumbency and draped for sterile surgery. The left knee was opened via incision. Vessels in the joint capsule were cauterized to prevent excess blood loss. The joint capsule was opened by incision with a #10 blade just below the patellar tendon. The patellar tendon was dislocated to expose the joint space. The fat pad was removed from the knee area to expose the tibial bone. The tibial canal was located and reamed using a 22-gauge needle. The space was widened using a drill with a 1.2-mm or 1.6-mm tungsten carbide drill bit. The bone tunnel was then dried and treated to simulate acute human PJI following primary arthroplasty. A stainless steel Kirschner wire implant 2.0-cm long with a 0.3-cm long top hook was placed in the tibial canal and the wound closed. Prior to closure of the superficial skin layer, 0.1 mL of 2×10^6^ planktonic methicillin-sensitive *S*. *aureus* SH1000 (CFU/rabbit) in saline was injected into the joint space. Closure of the joint space was performed with a continuous suture using 4–0 Vicryl (Ethicon). The skin was closed with a continuous subcutaneous (SC) 4–0 Vicryl suture and an additional continuous Vicryl suture over the outer skin layer. Proper insertion of the Kirschner wire implant into the tibial canal was verified in all rabbits by X-ray (Fig 2A).

### Rabbit PJI PLG0206 ex vivo, in vivo, and survival experiments

At 2 days post infection (2DPI), the joint space was reopened, debrided, and irrigated with 0.9% sodium chloride (Baxter). For the ex vivo model (PBS vehicle [irrigation and debridement (I&D) alone], *n* = 4; PLG0206, *n* = 5), infected implants were removed, washed three times in dPBS, and placed into 1 mg/mL PLG0206 in dPBS pH adjusted to 7.4 for 15 min then washed three times in PBS before being placed into PBST for sonication. For the short-term in vivo analysis (PBS vehicle [I&D alone], *n* = 8; PLG0206 15 min, *n* = 8; PLG0206 7.5 min, *n* = 7; PLG0206 5 min, *n* = 8), a non-survival surgery was performed. The joint space was opened, irrigated, and debrided and the joint capsule closed with 4–0 Vicryl sutures as described above. The debrided infected joint was treated intraoperatively with a 2 mL intra-articular injection of PLG0206 at 1 mg/mL in PBS pH 7.4 for 5, 7.5, and 15 min. Treatment was followed by a PBS rinse to precisely define the time of exposure. The animal was then euthanized with an intravenous overdose of Euthasol (100 mg/kg). Death was confirmed by loss of heartbeat and breath sounds. The implant and part of the proximal tibia were harvested and placed into PBST for sonication. Implant samples were sonicated using a Branson model #3510 sonicating water bath for 10 min. The proximal tibia was homogenized for 1 min using a TT10 basic Ultra-TURRAX homogenizer (IKA). For the survival analysis, rabbits (PBS vehicle [I&D alone], *n* = 7; cefazolin alone, *n* = 8; PLG0206 plus cefazolin, *n* = 8) received the implant and bacterial inoculation as described in the short-term in vivo experiments. At 2DPI, the infected joint was reopened, irrigated, debrided, and treated intraoperatively with 1 mg/mL PLG0206 for 15 min, after which the knee joint and skin layer were sutured closed as described above. Cefazolin (25 mg/kg) was administered twice daily for 5 days starting on 2DPI and stopping on 7DPI. Animals were monitored for signs of infection, improvement in PJI severity, and survival for up to 28 days. When an animal was sick or needed to be euthanized, animals were anesthetized with 20 mg/kg ketamine and 2 mg/kg xylazine. For euthanasia, animals were given an intravenous overdose of Euthasol (100 mg/kg). Death was confirmed by loss of heartbeat and breath sounds. The implant and a part of the tibia was collected postmortem and bacterial burden was determined by CFU analysis. All experiments were performed at least two independent times with similar results.

### Systemic administration of PLG0206 in a model of urinary tract infection

Murine surgical models were performed at TransPharm Preclinical Solutions, approved by the Institutional Animal Care and Use Committee, and in accordance with laws, regulations, and guidelines of the National Institutes of Health. Twenty-six fully immunocompetent BALB/c female mice aged 12 weeks (20–22 g) were challenged with *E*. *coli* (strain CFT073, TPPS1041) via intraurethral administration. The organism was grown for 15 h at 37°C on ambient atmosphere TSA plates supplemented with 5% sheep blood cells. The culture was aseptically swabbed and transferred to tubes of trypticase soy broth. The optical density was determined at 600 nm. Cultures were diluted to provide an inoculum of ~7.5 log_10_ CFU per mouse in a volume of 0.05 mL. Instillation of the bacterial challenge constituted time 0 h for the study. Inoculum densities were estimated before inoculation by optical density and confirmed after inoculation by dilution and back counts. On Day 0 at 0 h, mice were anesthetized through using isoflurane and brought to a surgical plane. Each mouse was challenged via transurethral injection (using a Hamilton syringe with a blunt-tipped 25 G needle) with ~7.5 log_10_ CFU per mouse in a dose volume of 0.05 mL. Challenge was performed in a BL2 surgical suite. The final count of the challenge inoculum demonstrated a delivered burden of 7.7 log_10_ organisms per mouse. Mice were treated with PLG0206 via IV administration or gentamicin via SC injection 2 h post challenge. PLG0206 was formulated in normal saline and administered via a single IV injection at the doses of 2 or 4 mg/kg following bacterial challenge on Day 0. Mice in the control group received a SQ injection of gentamicin on Day 0 at 2 h post challenge. Kidneys and bladders were harvested at 24 h post challenge to assess bacterial burden. The primary endpoint used to assess progress of the infection was mean bacterial burden per gram of kidney and urinary bladder tissue. At 24 h post challenge, mice were euthanized by CO_2_ asphyxiation and the kidneys and urinary bladder were aseptically removed, weighed, and homogenized using mini bead-beater. The resulting homogenate was serially diluted to 10^−7^ and plated in 5 μL spots onto TSA plates, supplemented with 5% sheep blood cells, and grown for 15 h at 37°C at ambient atmosphere. CFU were enumerated and the total bacterial burden per gram of kidney and urinary bladder tissue homogenate was calculated.

### Statistical analysis

In vitro biofilm and in vivo rabbit PJI analysis was performed using GraphPad Prism version 9. For the biofilm data, a two-sided unpaired Student’s *t*-test was used with significance set to *P*<0.05. For the rabbit PJI study, the raw data were tabulated within each time interval, and the mean and standard deviation were calculated for each endpoint by group. For the implant CFU data, a Kruskal–Wallis test with Dunn’s multiple comparison analysis for non-parametric data was performed with significance set to *P*<0.05. A Mann–Whitney test for non-parametric data was used for the ex vivo rabbit PJI results with significance set to *P*<0.05. For the CFUs from the rabbit survival study, a Mann–Whitney test comparing PBS vehicle (I&D alone) with the treatment groups, debridement and antibiotics with implant retention [DAIR] (PBS vehicle + cefazolin) and DAIR+ PLG0206, was used with significance notated as **P<0.01 and ***P<0.001. Survival rates were estimated using the Kaplan–Meier product-limit method, and survival time was defined as the days elapsed between treatment (Day 0) and death or end-of-study (Day 28). Cases in which rabbits lived past 28 days were right-censored. A log-rank test of equality of survival curves was performed to determine if survival curves differed significantly between the four groups.

## Results

### PLG0206 has broad-spectrum activity against MDR ESKAPEE pathogens

**Fig 3:**
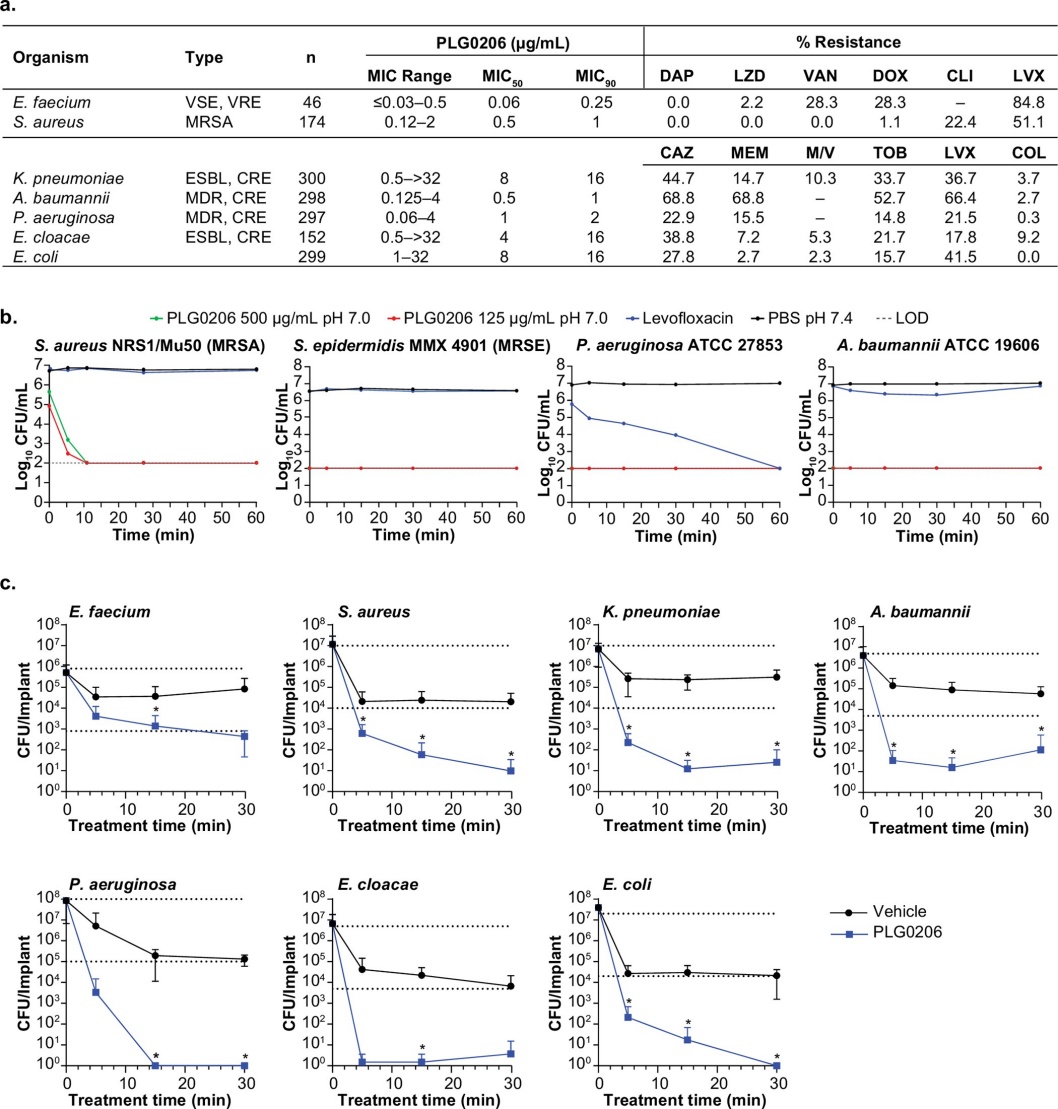
In vitro activity of the broad-spectrum engineered peptide antibiotic PLG0206. (a) MICs in mg/mL in sensitive and MDR bacterial strains. PLG0206 displays broad-spectrum activity and overcomes key resistant phenotypes. MDR where indicated is defined as resistance to ≥3 different antimicrobial classes/subclasses. ESBL screen based on ceftazidime MIC. (b) Bactericidal activity against planktonic bacteria. PLG0206 exhibits ≥3-log_10_ reduction in CFU/mL within 5 min of exposure. Levofloxacin was tested at 16-fold the MIC for each evaluated isolate. Levofloxacin MIC values were as follows: *S*. *aureus* NRS1 (16 mg/mL); *S*. *epidermidis* (0.25 mg/mL); *P*. *aeruginosa* (1 mg/mL); *A*. *baumannii* (1 mg/mL). (c) Bactericidal activity against biofilm bacteria grown on stainless steel wires. Top and bottom dotted line denote mean biofilm density and 3-log reduction, respectively. Data are mean from pooled data (standard deviation). Statistical comparisons (PLG0206 vs vehicle control) were performed with GraphPad Prism version 9 using Student’s *t*-test. *P<0.05. CAZ, ceftazidime; CFU, colony-forming units; CLI, clindamycin; COL, colistin; CRE, carbapenem-resistant; DAP, daptomycin; DOX, doxycycline; ESBL, extended spectrum beta-lactamase; LOD, limit of detection; LVX, levofloxacin; LZD, linezolid; MDR, multidrug resistant; MEM, meropenem; MIC, minimum inhibitory concentration; MRSA, methicillin-resistant *S*. *aureus*; MRSE, methicillin-resistant *S*.*epidermidis*; M/V, meropenem/vaborbactam; TOB, tobramycin; VAN, vancomycin; VRE, vancomycin-resistant enterococci; VSE, vancomycin-susceptible enterococci; -, no resistance breakpoint available.

Natural AMPs are primarily effective against Gram-negative bacteria, with limited activity against Gram-positive pathogens [23]. Initial studies suggested that PLG0206 had activity against select Gram-negative species as well as *S*. *aureus* [16,19]. We tested PLG0206 for broad-spectrum activity against a global panel of 1,267 MDR ESKAPEE clinical isolates. Broth culture susceptibility testing with PLG0206 demonstrated broad-spectrum activity against MDR Gram-positive ESKAPEE pathogens (Fig 3A). PLG0206 had a maximum MIC of 0.5 μg/mL against 46 vancomycin-sensitive/resistant *E*. *faecium* isolates, and 90% of those isolates (MIC_90_) had an MIC of 0.25 μg/mL. PLG0206 at a concentration of 2 μg/mL was sufficient to inhibit the growth of 156/174 methicillin-resistant *S*. *aureus* (MRSA) isolates exhibiting resistance to the important clinical antibiotics, clindamycin and levofloxacin.

Gram-negative ESKAPEE pathogens commonly carry resistance determinants for many clinically important antibiotics. Extended-spectrum beta-lactamases (ESBL) degrade important cephalosporins such as ceftazidime, and the *K*. *pneumonia*e carbapenemase inactivates both beta-lactams and carbapenems (e.g. meropenem). It is common for these strains to also be resistant to aminoglycosides (e.g. tobramycin), quinolones (e.g. levofloxacin), beta-lactam/beta-lactamase inhibitors (e.g. meropenem and vaborbactam), and the antibiotic of last resort, colistin. Fig 3A demonstrates that PLG0206 had potent activity against *A*. *baumannii* and *P*. *aeruginosa*, with MIC_90_ values of 1 and 2 μg/mL, respectively. Activity was lower for *K*. *pneumoniae* and *E*. *cloacae*, with an MIC_90_ of 16 μg/mL for both pathogens, as well as for an additional 299 *E*. *coli* isolates. Examination of the comparator antibiotic data in Fig 3A for the 1,346 Gram-negative isolates revealed that PLG0206 maintained activity against this large population of MDR clinical isolates.

### PLG0206 is rapidly bactericidal against MDR planktonic cells and biofilm

Although many clinically useful antibiotics are bactericidal, this generally requires significant exposure time to achieve the desired bactericidal effect, ≥3 log killing. AMPs are generally considered to be bactericidal; however, there are no approved members of this class in which killing is broad spectrum in nature [24]. PLG0206 was designed to overcome these limitations (Fig 1). We determined the planktonic time-kill kinetics of PLG0206 against a panel of Gram-positive and Gram-negative isolates which had PLG0206 MIC values of 1–8 μg/mL when tested in CAMHB (S2 Table). Bacterial cultures were exposed to either 125 or 500 μg/mL of PLG0206 for different time intervals and compared with the control bactericidal antibiotic levofloxacin. PLG0206 was rapidly bactericidal, exhibiting ≥3-log_10_ reduction in CFU/mL within 15 min of exposure for *S*. *aureus* (VISA), *Staphylococcus epidermidis* (MRSE), *P*. *aeruginosa*, and *A*. *baumannii* (Fig 3B). In comparison, levofloxacin-treated bacteria maintained constant CFU/mL, similar to vehicle-treated controls, across all treatment time points (Fig 3B). PLG0206 had rapid broad-spectrum bactericidal activity against each of these planktonic MDR ESKAPEE pathogens.

A limitation of current antibiotics is that they are only active against planktonic cells and exhibit little-to-no activity against biofilm cells [25–28]. Bacteria in biofilm have a high tolerance to antibiotics and this phenotype is, in part, based on a reduced metabolic state [28]. Based on its membrane-targeting mechanism of action, we hypothesized that PLG0206 would have rapid, broad-spectrum activity against biofilm bacteria. The mechanism of action for PLG0206 includes altering the permeability of bacterial membranes [29], which should allow activity and bacterial inactivation independent of bacterial metabolic state [13,30,31]. This was supported by our previous work demonstrating that PLG0206 could rapidly eliminate *S*. *aureus* biofilm [16]. To test the broad-spectrum biofilm activity of PLG0206, MDR ESKAPEE pathogens were selected from the clinical library used in the planktonic MIC screen.

PLG0206 demonstrated potent activity against MDR ESKAPEE biofilm pathogens (Fig 3C). *E*. *faecium* biofilms treated with PLG0206 for 30 min resulted in a 3.0-log_10_ reduction in bacterial burden compared with untreated controls. *S*. *aureus* biofilms treated with PLG0206 demonstrated a 4.3-log_10_ reduction in bacterial burden in 5 min. *K*. *pneumoniae* and *A*. *baumannii* biofilms resulted in a similar time-kill effect, with 4.5-log_10_ and 5.1-log_10_ reduction, respectively, in 5 min. *P*. *aeruginosa* biofilms were culture negative after 15 min of treatment with PLG0206 (7.9-log_10_ reduction). *E*. *cloacae* and *E*. *coli* biofilms were culture negative after 5 min (6.6-log_10_ reduction) and 30 min (7.6-log_10_ reduction) of PLG0206 treatment, respectively. These data suggest that PLG0206 is a potent antimicrobial agent to independently eliminate or reduce diverse bacterial biofilms without the use of additional antibiotic [32].

### PLG0206 has a low propensity for emergence of resistance

PLG0206 will be applied topically for the treatment of PJI using concentrations of 3 and 10 mg/mL, with an exposure time of 15 minutes. Therefore, the most appropriate model to examine resistance development is spontaneous mutation frequency (SMF), as opposed to long term exposure via serial passage. We conducted single-step spontaneous mutation frequency (SMF) experiments to assess development of resistance to PLG0206 for several Gram-positive and Gram-negative pathogens (Table 1). The agar dilution MIC for each isolate was determined, and then each was exposed to 4×, 8×, or 16× the agar dilution MIC of PLG0206 and incubated for 48 h before observing the plates for potential spontaneous mutants. Low SMF values of ≤10^−10^ to ≤10^−9^ were observed with four of the six *S*. *aureus* isolates above 4× the MIC, as well as for *S*. *epidermidis*, *E*. *faecalis*, and *E*. *faecium*. The two remaining *S*. *aureus* strains ATCC 29213 and NRS382 recorded SMF values of ≤10^−9^ at 16× the MIC. Mutant prevention concentration (MPC) values for PLG0206 were typically 16 to 32 μg/mL. For these Gram-positive isolates, none of the putative spontaneous mutant colonies demonstrated elevated PLG0206 MIC values after further evaluation by broth microdilution MIC testing.

**Table 1:** Spontaneous mutation frequency observed with PLG0206.

Organism	Isolate	Phenotype	Selection Concentration (μg/mL)	Fold MIC	Spontaneous Mutation Frequency	MPC (μg/mL)
*S*. *aureus*	ATCC 29213	MSSA QC	4	4	2.01E-07	>16
			8	8	4.54E-08	
			16	16	1.03E-09	
	NRS130	MSSA ERY^R^	8	4	1.76E-08	32
			16	8	1.18E-09	
			32	16	≤1.18E-09	
	NRS169	MSSA ERY^R^ CLI^R^	4	4	3.14E-08	16
			8	8	4.24E-09	
			16	16	≤8.47E-10	
	NRS1	MRSA VISA	8	4	≤1.98E-09	≤8
			16	8	≤1.98E-09	
			32	16	≤1.98E-09	
	NRS384	MRSA (USA300)	8	4	3.48E-08	32
			16	8	7.09E-10	
			32	16	≤7.09E-10	
	NRS382	MRSA (USA100)	4	4	2.39E-08	16
			8	8	2.84E-08	
			16	16	≤1.14E-09	
*S*. *epidermidis*	MMX 8740	MRSE	4	4	N/A	8
			8	8	≤1.22E-09	
			16	16	≤1.22E-09	
	MMX 8655	MRSE	4	4	N/A	8
			8	8	≤1.19E-09	
			16	16	≤1.19E-09	
*E*. *faecalis*	ATCC 700802	VanB VRE	4	4	≤2.94E-10	≤4
			8	8	≤2.94E-10	
			16	16	≤2.94E-10	
*E*. *faecium*	MMX 0485	VanA VRE	4	4	≤8.93E-10	≤4
			8	8	≤8.93E-10	
			16	16	≤8.93E-10	
*P*. *aeruginosa*	ATCC 27853	QC	8	4	8.00E-08	>32
			16	8	6.85E-08	
			32	16	1.31E-08	
	CDC 0241	IMP-1	8	4	8.72E-08	>32
			16	8	1.71E-07	
			32	16	1.14E-07	
	MMX 3025	CIP^R^	16	4	6.80E-08	64
			32	8	5.74E-09	
			64	16	≤8.20E-10	

CLI^R^, clindamycin-resistant; CIP^R^, ciprofloxacin-resistant; ERY^R^, erythromycin-resistant; IMP-1, metallo-β-lactamase; MIC, minimum inhibitory concentration; MPC, mutant prevention concentration; MRSA, methicillin-resistant *S*. *aureus*; MSSA, methicillin-susceptible *S*. *aureus*; MRSE, methicillin-resistant *S*. *epidermidis*; N/A, not available; QC, quality control; VISA, vancomycin-intermediate *S*. *aureus*; VRE, vancomycin-resistant enterococci.

The calculated SMF and corresponding MPC values for the three *P*. *aeruginosa* isolates are also shown in Table 1. For isolates ATCC 27853 and CDC 0241, spontaneous mutants were readily selected at 4×, 8×, and 16× the MIC of PLG0206, resulting in SMF ≥10^−8^. For the remaining isolate MMX 3025, the SMF was ≤10^−9^ for PGL0206 at 8× and 16× the MIC with an MPC of 64 μg/mL. In contrast to the Gram-positive bacteria described above, these spontaneous mutants of *P*. *aeruginosa* demonstrated broth microdilution MIC values that were ≥4-fold higher than that of the parent isolate (Table 2). However, the highest broth microdilution MIC values of these spontaneous mutants (32 μg/mL) were 100 to 300 times lower than the expected clinical exposures of 3 and 10 mg/mL for the local application of PLG0206 that are being explored to treat PJI (ClinicalTrials.gov Identifier: NCT05137314). In the case of *P*. *aeruginosa* CDC 0241, some of the PLG0206 spontaneous mutants also demonstrated elevated colistin MIC values (Table 2), though this should not affect future clinical outcomes since colistin-resistant Gram-negative infections are rare in PJI. However, it does warrant further investigation into mutations present in these spontaneous mutants and whether they share resistance mechanisms that are known for colistin.

**Table 2:** Broth microdilution MIC values (μg/mL) for *P*. *aeruginosa* spontaneous mutants selected with PLG0206 relative to parent strains.

Isolate	Selection Concentration in μg/mL-fold MIC	Designation*	PLG0206	IMP	COL	AZT	LVX	GM	CAZ	TZP^†^
ATCC 27853	N/A	Parent	0.5	1	0.25	4	1	1	2	4
	8–4×	P-8-103-A	1	2	0.25	4	0.5	1	2	4
		P-8-103-B	1	1	0.25	4	0.5	1	2	4
		P-8-103-1	1	1	0.25	4	0.5	1	2	4
		P-8-103-2	1	1	0.25	4	0.5	1	2	4
		P-8-103-3	1	1	0.25	4	0.5	1	2	4
	16–8×	P-16-103-A	2	1	0.25	4	1	1	2	4
		P-16-103-B	2	2	0.25	8	1	2	2	8
		P-16-103-1	1	1	0.25	4	0.5	1	4	8
		P-16-103-2	1	1	0.25	4	0.5	1	2	4
		P-16-103-3	1	1	0.25	4	0.5	1	2	4
	32–16×	P-32-103-A	**8**	1	0.25	4	1	1	2	4
		P-32-103-B	**4**	1	0.25	8	1	1	2	4
		P-32-103-1	1	1	0.25	8	0.5	1	*16*	8
		P-32-103-2	1	1	0.25	4	0.5	1	2	4
		P-32-103-3	1	2	0.25	8	1	2	*16*	*16*
CDC 0241	N/A	Parent	0.5	>32	0.25	16	16	32	>32	128
	8–4×	P-8-10166-A	**4**	>32	0.5	16	32	64	>32	128
		P-8-10166-B	**8**	>32	*1*	16	32	64	>32	128
		P-8-10166-1	1	>32	0.5	16	32	64	>32	128
		P-8-10166-2	**2**	>32	0.5	16	32	64	>32	128
		P-8-10166-3	**2**	>32	0.5	16	32	64	>32	128
	16–8×	P-16-10166-A	**16**	>32	*1*	16	32	64	>32	128
		P-16-10166-B	**16**	>32	*2*	16	32	64	>32	128
		P-16-10166-1	1	>32	0.5	16	32	64	>32	128
		P-16-10166-2	**2**	>32	0.5	16	32	32	>32	128
		P-16-10166-3	**2**	>32	0.5	16	32	64	>32	128
	32–16×	P-32-10166-A	**16**	>32	*4*	32	32	64	>32	128
		P-32-10166-B	**32**	>32	*4*	32	32	64	>32	128
		P-32-10166-1	2	>32	0.5	16	32	64	>32	128
		P-32-10166-2	**4**	>32	*2*	16	32	64	>32	128
		P-32-10166-3	**4**	>32	*1*	32	32	64	>32	128
MMX 3025	N/A	Parent	0.5	1	0.25	16	16	32	8	>256
	16–4×	P-16-3025-A	**8**	1	0.25	16	16	32	4	>256
		P-16-3025-B	**4**	1	0.25	16	16	32	8	>256
		P-16-3025-1	1	1	0.25	16	16	32	4	>256
		P-16-3025-2	**2**	1	0.25	16	16	32	4	>256
		P-16-3025-3	1	1	0.25	16	16	32	4	>256
	32–8×	P-32-3025-A	**8**	1	0.25	16	32	32	8	>256
		P-32-3025-B	**8**	1	0.25	16	32	32	8	>256
		P-32-3025-1	**4**	1	0.5	16	32	32	8	>256
		P-32-3025-2	**2**	1	0.25	16	32	32	8	>256
		P-32-3025-3	**8**	1	0.25	16	32	32	8	>256

**Bold** cells indicate ≥4-fold increase in MIC for the selection agent relative to that observed with the parent; *Italic* cells indicate ≥4-fold increase in MIC for an agent not used for selection relative to that observed with the parent.

* Designation includes drug (P designates PLG0206), selection concentration, MMX strain number, and an alphanumeric designation of patched smear (alphabetical) or colony (numeric) tested.

^†^ MIC reported for TZP reflects piperacillin concentration (tazobactam tested at constant concentration of 4 μg/mL).

AZT, aztreonam; CAZ, ceftazidime; COL, colistin; GM, gentamicin; IMP, imipenem; LVX, levofloxacin; MIC, minimum inhibitory concentration; N/A, not applicable; TZP, piperacillin/tazobactam.

After observing the isolation of *P*. *aeruginosa* spontaneous mutants after exposure to low (8, 16, or 32 μg/mL) concentrations of PLG0206, we conducted a SMF study in which *P*. *aeruginosa* CDC 0241 and two other *P*. *aeruginosa* isolates were exposed to 500 μg/mL PLG0206 for 20 min, representing a more clinically relevant concentration and treatment time. After sampling the culture for viable cells, the PLG0206 treated cultures were washed to remove excess drug, followed by plating on agar containing either 4, 8, or 16-fold the MIC of PLG0206 as is conducted in a traditional SMF assay. Table 3 shows that this 20-min exposure resulted in a 4.0–6.8 log_10_ kill for the three *P*. *aeruginosa* strains evaluated. Washing these cells to remove excess drug further reduced the culture density, resulting in a range of 20–32,000 CFUs actually applied to the spontaneous mutation plates. No spontaneous mutant colonies were selected for the three evaluated isolates. We conclude that the high degree of killing observed in vitro during a 20-min exposure to 500 μg/mL of PLG0206 reduces the cell population such that selection of spontaneous mutants is highly unlikely.

**Table 3:** Spontaneous mutant selection of *P*. *aeruginosa* after exposure to 500 μg/mL PLG0206 for 20 minutes.

Isolate	Culture treatment	Mean CFU/ml	CFU applied to SMF plates	Δlog_10_ relative to initial PBS cell suspension	SMF* colonies
ATCC 27853	Initial cell suspension in PBS	4.5E+09	-	-	-
	After 20 min. exposure to 500 μg/ml PLG0206	4.6E+05	-	-4.0	-
	After washing cells prior to SMF plating	1.6E+05	-	-4.5	-
	Spontaneous mutation selection^†^	-	3.2E+04	-	0
CDC 0241	Initial cell suspension in PBS	5.5E+09	-	-	-
	After 20 min. exposure to 500 μg/ml PLG0206	5.5E+05	-	-4.0	-
	After washing cells prior to SMF plating	5.0E+02	-	-7.1	-
	Spontaneous mutation selection	-	1.00E+02	-	0
MMX 3025	Initial cell suspension in PBS	7.5E+09	-	-	-
	After 20 min. exposure to 500 μg/ml PLG0206	1.3E+03	-	-6.8	-
	After washing cells prior to SMF plating	1.0E+02	-	-7.9	-
	Spontaneous mutation selection	-	2.00E+01		0

* Spontaneous mutation frequency.

^**†**^ Spontaneous mutants were selected on RPMIA containing either 4, 8, or 16-fold the PLG0206 MIC value for that isolate.

CFU, colony-forming unit; PBS, phosphate-buffered saline; SMF, spontaneous mutation frequency.

### PLG0206 is an effective treatment for biofilm-associated periprosthetic joint infection

Natural AMPs evolved to work against defined bacterial targets in particular environments [12]. Environmental fluctuations (pH, ion concentrations) can adversely affect activity; this has been a factor limiting their successful clinical development. There has also been a poor translation from in vitro activity against pathogens to in vivo efficacy against infections. This has been attributed to their contextual activity based on environmental factors like salinity, serum, and pH as well as low plasma stability due to their susceptibility to host protease activity and rapid hepatic and renal clearance [12–14]. PLG0206 was designed to allow it to overcome the in vivo limitations that have plagued natural AMPs. Based on the rapid and broad-spectrum biofilm activity observed in vitro, we hypothesized that PLG0206 could be used to treat biofilm-associated infections in a clinical setting. Biofilm-based PJI can be a catastrophic complication of joint arthroplasty, one of the most common major surgical procedures. The high tolerance of biofilms to antibiotics makes it difficult to treat PJI medically, with surgery remaining the current standard-of-care treatment. DAIR is the clinically preferred treatment approach, but biofilm antibiotic tolerance results in failure rates of around 60% [33,34].

To test the efficacy of intraoperative PLG0206 as a biofilm-specific adjuvant in a single DAIR procedure, a New Zealand white rabbit methicillin-susceptible *S*. *aureus* (MSSA) PJI surgical model was used. *S*. *aureus* is the most common organism that causes PJI, and MSSA are more pathogenic than MRSA infections in rabbits [35]. In this model, the right knee was exposed using a lateral arthrotomy and a Kirschner wire implant was placed intra-articular into the intramedullary space and inoculated with *S*. *aureus*. Proper insertion of the Kirschner wire implant into the tibial canal was verified by X-ray (Fig 2A). On post-operative and post-infection Day 2, the knee was surgically exposed, and implants were treated with PLG0206 or control (PBS vehicle: I&D alone) either ex vivo or in vivo with a DAIR procedure. Following ex vivo treatment of the MSSA-infected implants, PLG0206-treated dose groups were significantly different compared to vehicle controls, with a 3.3 log_10_ reduction in bacterial burden following PLG0206 treatment (S1 Fig). Directly following I&D, 1 mg/mL PLG0206 for 5, 7.5, and 15 min had a similar decrease (2.2-log_10_ reduction) in bacterial load as compared to I&D-only PBS vehicle controls, and the CFU reduction was treatment time dependent (Fig 2B).

These short-term surgical exposure results indicated that 1 mg/mL PLG0206 treatment is effective at reducing bacterial biofilm in a PJI intraoperative model at all exposure time points tested. Next, we tested the efficacy of PLG0206 in a long-term survival study after a single DAIR procedure. In this rabbit model, PJI is fatal when antibiotics are discontinued following DAIR. After I&D, the joint space was treated for 15 min with 1 mg/mL PLG0206 or PBS vehicle intraoperatively. Following closure, cefazolin, a standard-of-care antibiotic for MSSA PJI, was administered for 5 days, and then discontinued, and long-term animal health and survival were monitored. Rabbits treated with only a DAIR procedure followed by cefazolin exhibited 100% mortality by 14 days post infection (Fig 2C). Several animals (75%) had culture-negative implants upon harvest. However, the majority of animals (88%) had developed a large abscess in the knee joint area, indicating an ongoing active infection. In contrast, treatment with 1 mg/mL PLG0206 in combination with a DAIR procedure resulted in prolonged survival (63%) (Fig 2C) and a 2.5-log_10_ reduction in bacterial burden. Several rabbits had culture-negative implants (38%) and only 25% had a small abscess upon necropsy (S2 Fig). The reduction of bacteria in a brief time period from the control vehicle was expected as this is the irrigation and debridement portion of the procedure. The control group gives a measure of how much bacteria is removed by irrigation and debridement alone. Similar observations have been reported previously in the literature [36,37], and provide additional evidence that a thorough debridement is a key principle in treatment of implant associated infections.

### PLG0206 is an effective intravenous treatment for a urinary tract infection

After demonstrating in vitro and animal model efficacy of local application of PLG0206 against MDR infections, we sought to determine the efficacy of systemic administration of PLG0206. Systemic administration of unmodified peptides (e.g. IV injection) has generally been challenging due to a short half-life resulting from rapid degradation by proteolytic enzymes in blood plasma and rapid removal from the circulation by the liver (hepatic clearance) and kidneys (renal clearance) [38]. We tested if the design of PLG0206 would allow it to overcome these in vivo limitations that have limited natural AMPs. Based on the rapid and broad-spectrum activity observed in vitro against uropathogens, we hypothesized that PLG0206 could be used systemically to treat urinary tract infections, which are one of the most commonly reported infections. Fully immunocompetent BALB/c mice were challenged with *E*. *coli* (strain CFT073, TPPS 1041) via intraurethral administration. Mice were treated with PLG0206 via IV administration or gentamicin via SC injection 2 h following challenge. Kidneys and bladders were harvested at 24 h post challenge to assess bacterial burden (S3 Fig). The lower dose of PLG0206 (2 mg/mL) reduced CFU burden from 7.5 log_10_ CFU to the limit of detection in the kidneys and the bladder in 5 out 6 mice, at 24 h. The higher dose of PLG0206 (4 mg/mL) reduced CFU burden from 7.5 log_10_ CFU to the limit of detection in the kidney in 6 out of 6 mice and the bladder in 4 out of 6 mice, at 24 h. In this exploratory study, the data indicate that PLG0206 can be an effective IV treatment in this murine urinary tract infection model.

## Discussion

The clinical pipeline of new antimicrobials is limited, especially in development pathways trying to demonstrate the clinical impact of non-traditional antibacterial approaches [3]. The rational design of PLG0206 has overcome many of the limitations of traditional antibiotics (e.g. resistance and lack of anti-biofilm activity) and AMPs, and we are currently developing PLG0206 for the treatment of biofilm infections caused by MDR bacteria. Using a large clinical isolate library of ESKAPEE pathogens, we demonstrated that PLG0206 possessed rapid, bactericidal, broad-spectrum activity against both Gram-positive and Gram-negative MDR pathogens in both planktonic and biofilm modes of growth. This contrasts with other antibiotics where there is a loss of activity to bacterial biofilms as compared with planktonic cells [25–28]. SMF studies revealed that Gram-positive pathogens did not produce spontaneous mutants with elevated PLG0206 MIC values, whereas *P*. *aeruginosa* did. However, exposing *P*. *aeruginosa* to a clinically relevant concentration of PLG0206 revealed that the strong bactericidal properties greatly reduced the cell numbers, making the isolation of spontaneous mutants highly unlikely. Gram-negative bacteria arguably have more ways to develop antibiotic resistance (e.g. outer membrane changes, many efflux pumps, etc.) than Gram-positive bacteria, and the mechanism of resistance to PLG0206 is under further study for *P*. *aeruginosa*. Other antimicrobial chemotherapeutic agents are unable to independently eliminate persistent biofilms [39,40]. This high antibiotic tolerance of biofilms makes medical device- and implant- associated infections, including PJI, difficult to treat with standard antibiotics. In contrast, in a large rabbit PJI animal model, PLG0206 maintained the biofilm-associated activity observed in vitro with no obvious toxicity, and for the first time allowed animals prolonged survival in a single DAIR procedure following *S*. *aureus* infection.

Another major limitation of AMPs includes systemic toxicity and poor PK activity along with a lack of systemic activity. Specifically, peptide-based antibacterials have been associated with a high incidence of nephrotoxicity and neurotoxicity associated with IV administration [41]. In a murine uropathogenic *E*. *coli* urinary tract infection model, systemically administered PLG0206 was effective at reducing bacterial loads in both the bladder and kidneys comparable to the antibiotic control. In a first-in-human IV-administered study, PLG0206 was safe and well tolerated with predictable linear PK with a median terminal half-life (t_½_) ranging from 6.5 to 11.2 h when administered at single IV doses ranging from 0.05 to 1 mg/kg [42]. These findings provide strong evidence to support continuing clinical development of PLG0206 for MDR infections. Finally, the results obtained with PLG0206 as a prototypical and successful example from the challenging AMP class validate that rational design of peptides can be used to overcome the challenges associated with difficult drug targets.

## Supporting information

S1 Table Impact of broth basal medium and different agars on the agar dilution MIC of PLG0206. MIC, minimum inhibitory concentration; RPMI, Roswell Park Memorial Institute 1640.
(PDF)

S2 Table Broth microdilution MIC values (μg/mL) for *P*. *aeruginosa* spontaneous mutants selected with PLG0206 relative to parent strains. ^1^ Broth microdilution MIC testing in CAMHB per CLSI [20,21]; ^2^ Quality control MIC range [20,21]. CAMHB, cation-adjusted Mueller-Hinton Broth; MIC, minimum inhibitory concentration; MRSE, methicillin-resistant *S*. *epidermidis;* VISA, vancomycin-intermediate *S*. *aureus*.
(PDF)

S1 Fig Bacterial burden of infected implants. Ex vivo analysis of PLG0206 was performed by removing the infected implant at 2 days post infection and treating with 1 mg/mL PLG0206 for 15 min. Compared with implants from PBS vehicle (I&D alone) (*n* = 4), implants treated with PLG0206 (*n* = 5) displayed significantly reduced *S*. *aureus* biofilm burden. Data are mean (standard deviation). Mann-Whitney statistical analysis was performed using GraphPad Prism version 9. *P = 0.016. CFU, colony-forming units; I&D, irrigation and debridement; PBS, phosphate-buffered saline.
(EPS)

S2 Fig Bacterial burden of infected joints. Treatment with PLG0206 in combination with cefazolin prolonged animal survival. Rabbits (PBS vehicle [I&D alone], *n* = 7; cefazolin, *n* = 8; cefazolin + PLG0206, *n* = 8) were given PJI by placement of a Kirschner wire implant into the proximal tibia followed by an inoculation of MSSA *S*. *aureus* (SH1000) into the knee joint space. Two days post infection, the knee joint was irrigated and debrided. The infected implant was subjected to long-term survival experiments using 1 mg/mL PLG0206. Bacterial burden from survival experiments: PLG0206 plus cefazolin treatment resulted in a 2.5 log_10_ reduction in bacterial burden whereas cefazolin treatment alone was ineffective, with only a 1.2 log_10_ reduction. Data are mean (standard deviation). Mann-Whitney statistical analysis was performed using GraphPad Prism version 9. **P<0.01, ***P<0.001. CFU, colony-forming units; I&D, irrigation and debridement; MSSA, methicillin-susceptible *S*. *aureus*; PBS, phosphate-buffered saline; PJI, periprosthetic joint infection.
(EPS)

S3 Fig Bacterial burden of infected urinary tract. Treatment with the lower dose of PLG0206 (2 mg/mL) reduced CFU burden from 7.5 log_10_ CFU to the limit of detection in the kidneys and the bladder in 5 out of 6 mice, at 24 h. The higher dose of PLG0206 (4 mg/mL) reduced CFU burden from 7.5 log_10_ CFU to the limit of detection in the kidney in 6 out of 6 mice and the bladder in 4 out of 6 mice, at 24 h. CFU, colony-forming units.
(EPS)

S1 Dataset Data supporting results shown in Figs 2B, 2C and 3C, and S1 and S2 Figs. (PDF)

S2 Dataset Data supporting results shown in minimal inhibitory concentration studies (Table 2, Fig 3A, and S1 and S2 Tables). (XLSX)

S3 Dataset Data supporting results shown in Tables 1 and 3, Fig 3B, and S3 Fig. (XLSX)
