# Supplementary material for: Engineered peptide PLG0206 overcomes limitations of a challenging antimicrobial drug class
Source: PLoS One. 2022 Sep 16;17(9):e0274815. doi: 10.1371/journal.pone.0274815 (PMC9481017; doi:10.1371/journal.pone.0274815)
Supplement: S1 Table — MIC, minimum inhibitory concentration; RPMI, Roswell Park Memorial Institute 1640. (PDF) [file pone.0274815.s001.pdf]

Organism	MIC (µg/mL) in agar media (triplicate values)			
	**Mueller-Hinton Agar**	**RPMI Bacto Agar**	**RPMI Noble Agar**	**RPMI EEO Agarose**
S. aureus ATCC 29213	>64	>64	16	0.5
	>64	>64	16	0.25
	>64	>64	16	0.25
E. coli ATCC 25922	>64	>64	16	1
	>64	>64	16	1
	>64	>64	16	1
P. aeruginosa ATCC 27853	>64	>64	32	2
	>64	>64	32	2
	>64	>64	32	2

S1 Table. Impact of broth basal medium and different agars on the agar dilution MIC of PLG0206

MIC, minimum inhibitory concentration; RPMI, Roswell Park Memorial Institute 1640
