# Supplementary material for: Engineered peptide PLG0206 overcomes limitations of a challenging antimicrobial drug class
Source: PLoS One. 2022 Sep 16;17(9):e0274815. doi: 10.1371/journal.pone.0274815 (PMC9481017; doi:10.1371/journal.pone.0274815)
Supplement: S2 Table — 1 Broth microdilution MIC testing in CAMHB per CLSI [20,21]; 2 Quality control MIC range [20,21]. CAMHB, cation-adjusted Mueller-Hinton Broth; MIC, minimum inhibitory concentration; MRSE, methicillin-resistant S. epidermidis; VISA, vancomycin-intermediate S. aureus. (PDF) [file pone.0274815.s002.pdf]

Organism Name	MMX No.	Alternate No.	Type	MIC (µg/ml)[1]	
				PLG0206	Levofloxacin
S. aureus	1723	NRS1 (Mu50)	VISA	4	8
S. epidermidis	4901	MMX 4901	MRSE	1	0.25
P. aeruginosa	0103	ATCC 27853	Sensitive to most antibiotics	8	1 (0.5-4)[2]
A. baumannii	1630	ATCC 19606	Sensitive to most antibiotics	4	1

S2 Table. Broth microdilution MIC values (µg/mL) for *P. aeruginosa* spontaneous mutants selected with PLG0206 relative to parent strains

[1] Broth microdilution MIC testing in CAMHB per CLSI (39, 40); [2] Quality control MIC range (39, 40)

CAMHB, cation-adjusted Mueller-Hinton Broth; MIC, minimum inhibitory concentration; MRSE, methicillin-resistant *S. epidermidis;* VISA, vancomycin-intermediate *S. aureus*
