# Supplementary material for: Engineered peptide PLG0206 overcomes limitations of a challenging antimicrobial drug class
Source: PLoS One. 2022 Sep 16;17(9):e0274815. doi: 10.1371/journal.pone.0274815 (PMC9481017; doi:10.1371/journal.pone.0274815)
Supplement: S1 Dataset — (PDF) [file pone.0274815.s006.pdf]

Figure S1 Ex vivo Rabbit

PBS Vehicle	Treated
26600	1
106600	1
13600	20
180000	20
	170

Figure 2B In vivo rabbit

PBS Vehicle	15 min	7.5 min	5 min
5000	410	10	100
1600000	5600	1750	1060
36666	800	30	60
7100	1666	7666	120
60000	1290	1140	14700
16666	1390	10	910
12000	550	400	8333
4666	440		6000

Figure 2C Rabbit survival

Days	PBS Vehicle	DAIR (I&D + Cefazolin)	DAIR + PLG0206
2	1		
2	1		
4	1		
7	1		
7	1		
8	1		
8	1		
6			
11		1	
12		1	
9		1	
14		1	
11		1	
11		1	
10		1	
10		1	
28			0
28			0
28			0
7			1
28			0
28			0
11			1
5			1

Figure S2 Rabbit survival CFU

PBS Vehicle	PLG0206	Cefazolin	Cefazolin+PLG0206
2000	700000	1	20
200000	35667	1	1
13000	70000	1	50
7600	120000	80	20
22667	25667	1	10
4667	11667	1	1
10333	14333	16667	1
	6000	1	900

Figure 3C In vitro biofilms

E. faecium	Time (min)	Vehicle																	
	**0**	20000	330000	170000	160000	1100000	400000	83333	100000	230000	170000	570000	2700000	30000	400000	1000000	1500000	200000	110000
	**5**	4667	20000	1333	40000	100000	100000	2333	3333	5333	10000	36667	270000	13333	13333	10000	2667	333	333
	**15**	13333	5333	20000	300000	70000	97000	2667	3333	3333	3000	46667	70000	16667	3667	4000	2000	4000	1000
	**30**	5000	20000	16667	150000	93333	33333	1333	2667	23333	200000	10000	66667	800000	30000	1000	33333	1333	10000
	**Time (min)**	**PLG0206**																	
	**0**	20000	330000	170000	160000	1100000	400000	83333	100000	230000	170000	570000	2700000	30000	400000	1000000	1500000	200000	110000
	**5**	1200	1090	1010	3500	2700	4080	1010	530	62	640	3800	20000	400	890	2500	30000	890	390
	**15**	450	1500	580	1220	300	13300	300	1090	120	400	690	1300	820	600	530	620	450	800
	**30**	210	180	230	420	70	20	550	310	500	620	1300	170	400	380	100	130	980	1300

S. aureus	Time (min)	Vehicle																	
	0	200000	18000000	10000000	13000000	830000	9300000	47000000	60000000	20000000	10000	470000	630000	500000	12000000	15700000	470000	120000	2900000
	5	10000	23333	13333	10000	1667	1000	5667	60000	16667	5000	4000	10000	1333	2000	667	6667	167000	40000
	15	26667	43333	33333	9000	13333	10000	16667	16667	10000	10000	4667	1000	1333	1333	7333	13333	167000	53333
	30	20000	13333	30000	8667	1333	16667	1667	4667	43333	1667	3333	4667	667	4000	10000	13333	130000	60000
	Time (min)	PLG0206																	
	0	200000	18000000	10000000	13000000	830000	9300000	47000000	60000000	20000000	10000	470000	630000	500000	12000000	15700000	470000	120000	2900000
	5	110	270	50	1	1	1	2700	570	1300	1	1	1	1	3333	1333	133	1333	1
	15	1	1	1	1	1	1	470	60	510	1	1	1	1	1	1	1	1	1
	30	1	1	1	1	1	1	40	20	100	1	1	1	1	1	1	1	1	1

K. pneumoniae	Time (min)	Vehicle																	
	0	8300000	21000000	8300000	2000000	4000000	15000000	4700000	3300000	4700000	6700000	7600000	5300000	700000	1470000	3300000	3300000	5000000	21000000
	5	270000	930000	500000	100000	170000	53333	130000	500000	100000	60000	86667	167000	130000	267000	530000	300000	170000	230000
	15	130000	430000	630000	140000	130000	76667	170000	90000	270000	83333	167000	100000	200000	300000	530000	370000	230000	170000
	30	200000	300000	83333	230000	120000	170000	300000	96667	230000	83333	50000	200000	470000	270000	430000	330000	300000	1700000
	Time (min)	PLG0206																	
	0	8300000	21000000	8300000	2000000	4000000	15000000	4700000	3300000	4700000	6700000	7600000	5300000	700000	1470000	3300000	3300000	5000000	21000000
	5	80	1	1	10	30	400	1200	1020		160	50	30	60	30	20	600	1	100
	15	20	10	1	1	1	1	1	30	70	1	30	1	10	40	1	1	1	1
	30	320	1	1	1	1	1	1	1	1	60	1	1	1	60	1	1	1	1

A. baumannii	Time (min)	Vehicle																	
	0	1100000	870000	2300000	970000	770000	1200000	5700000	1000000	800000	1800000	970000	1500000	1400000	500000	3300000	28000000	6000000	12000000
	5	170000	20000	470000	23333	570000	270000				4333	50000	120000	6000	33333	5667	56667	106000	230000
	15	60000	16667	567	23333	23333	10000	66667	70000		300000	260000	20000	36667	2000	30000	400000	63333	130000
	30	76667	36667	14333	53333	16667	20000	36667	26667	73333	36667	66667	300000	1667	20000	1000	70000	80000	120000
	Time (min)	PLG0206																	
	0	1100000	870000	2300000	970000	770000	1200000	5700000		800000	1800000	970000	1500000	1400000	500000	3300000	28000000	6000000	12000000
	5	10	20	1	10	1	1	40	1	80	20	300	10	1	1	1	80	1	50
	15	10	10	1	1	10	10				10	100	80	1	1	1	1	1	1
	30	1	1	1	1	1	10	1	1	1	2010	1	1	1	1	1	1	10	20

P. aeruginosa	Time (min)	Vehicle																	
	0	8000000	6600000	37700000	19000000	39000000	28000000	2.4E+08	1E+08	90000000	2.2E+08	1.1E+08	1.2E+08	9000000	10000000	11000000	12000000	8000000	9000000
	5	330000	1370000	330000	730000	36667	16667	57000000	160000	100000	400000	670000	470000	600000	490000	320000	600000	250000	
	15	16667	50000	70000	93333	140000	330000	200000	100000	230000	700000	230000	170000	90000	120000	110000	500000		370000
	30	26667	120000	160000	180000	167000	120000	200000	230000	37000	53000	70000	230000	200000	320000	500000	670000	350000	400000
	Time (min)	PLG0206																	
	0	11700000	6600000	37700000	19000000	39000000	28000000	2.4E+08	1E+08	90000000	2.2E+08	1.1E+08	1.2E+08	9000000	10000000	11000000	12000000	8000000	9000000
	5	1	1	1	40000	1	1	1	1	10	1	70	20	10	30	70	1	1	1
	15	1	1	1	1	1	1	1	1	1	1	1	1	1	1	1	1	1	1
	30	1	1	1	1	1	1	1	1	1	1	1	1	1	1	1	1	1	1

E. cloacae	Time (min)	Vehicle																	
	0	160000	210000	370000	6000	11000	200000	1000000	32000000	42000000	1000000	9000000	7700000	830000	1100000	2100000	6000000	7700000	8000000
	5	1	333	333	1	1	1	400000	230000	30000	4333	20000	6000	3667	1333	3333	43333	4667	10000
	15	1	1	1	1	1	1	93333	66667	56667	10000	26667	13000	3333	1333	4333	36667	20000	66667
	30	1	1	1	1	1	1	1	1	1	2333	2000	1667	1667	6000	10333	46667	43333	5667
	Time (min)	PLG0206																	
	0	160000	210000	370000	6000	11000	200000	1000000	32000000	42000000	1000000	9000000	7700000	830000	1100000	2100000	6000000	7700000	8000000
	5	1	1	1	1	1	1	1	1	1	1	1	1	1	1	1	1	10	1
	15	1	1	1	1	1	1	1	1	1	1	1	1	1	1	1	1	10	1
	30	1	1	1	1	1	1	1	1	1	1	1	1	50	1	1	1	1	1

E. coli	Time (min)	Vehicle																	
	0	1500000	5300000	700000	247000	1200000	1300000	100000	4700000	7700000	5300000	7700000	15000000	50000000	57000000	1.07E+08	77000000	2.5E+08	1.16E+08
	5	10000	46667	10000	2333	2667	36667	4000	6667	6667	15000	17000	66000	130000	100000	20000	333	6666	6666
	15	1333	2000	30000	110000	13000	66667	10000	8000	2333	17000	73000	10000	100000	13000	6667	10000	53333	16667
	30	77000	43000	37000	4333	23333	23333	2000	33333	33333	2000	1333	6667	36667	5000	20000	1333	13333	23333
	Time (min)	PLG0206																	
	0	1500000	5300000	700000	247000	1200000	1300000	100000	4700000	7700000	5300000	7700000	15000000	50000000	57000000	1.07E+08	77000000	2.5E+08	1.16E+08
	5	1	1	1	1	1	1	333	1667	1333	1	10	1	1	1	120	1	100	200
	15	1	1	1	1	1	1	1	1	1	1	1	1	1	1	1	1	100	200
	30	1	1	1	1	1	1	1	1	1	1	1	1	1	1	1	1	1	1

0
